# Microstructural Characterization of Cobalt-Tungsten Coated Graphite Fibers

**DOI:** 10.6028/jres.100.048

**Published:** 1995

**Authors:** N. S. Wheeler

**Affiliations:** University of Virginia, Dept. of Materials Science and Engineering, Charlottesville, VA 22906; National Institute of Standards and Technology, Gaithersburg, MD 20899-0001

**Keywords:** carbon, cobalt, composite, graphite, TEM, XRD, tungsten

## Abstract

The present research concerns an electrodeposited cobalt-tungsten alloy coating on graphite fibers. Annealed and unannealed coated fibers were analyzed by scanning electron microscopy/energy dispersive x-ray spectroscopy (SEM/EDS), x-ray diffraction (XRD), and transmission electron microscopy (TEM). The mole fraction of tungsten in the as-deposited cobalt-tungsten coating was found to be (7.10 ± 0.82) %, and the crystalline lattice was determined to be hexagonal close-packed. Note: The uncertainties reported here are expanded uncertainties (i.e., 2 standard deviation estimates). After annealing at 1100 °C for 1.5 h, the lattice was found to be a cobalt-tungsten-carbon (Co-W-C) alloy with a face centered cubic lattice. The mole fraction of tungsten in the annealed coating was shown to be (5.30 ± 0.66) %. XRD analysis revealed that the annealed coating contained regions having three slightly different lattice parameters. The lattice parameters in the three regions of the Co-W-C alloy coating corresponded to a mole fraction of carbon of 0.66 %, 0.40 %, and 0.19 % (± 0.18 %). These compositions are discussed with respect to the Co-W-C phase diagram. Various tungsten carbide species were identified in the coating and fiber, and a network of overlapping WC crystals was found at the coating-fiber interface. This network appeared to serve as a protective barrier to extend the lifetime of the fibers at elevated temperatures.

## 1. Introduction

Use of graphite-nickel metal matrix composites for structural purposes, although attractive from a strength-to-density perspective, has not been realized due to the catastrophic high temperature interdiffusion of these species [1]. The electrodeposited Co-W alloy characterized in this study has been shown to be effective as a diffusion barrier coating on graphite fibers in a nickel matrix for up to 800 °C for as long as 24 h [2–4]. This represents a 700 % increase in the life of the fibers at this temperature. Exposure to higher temperatures or for longer times results in the interdiffusion problems mentioned above for graphite-nickel composites, although the severity is moderated by the alloy coating [2,3]. For example, at 1100 °C, the lifetime of the fibers was extended by approximately 500 % [3]. The present study was undertaken to determine the microstructure of the coated fibers after annealing at 1100 °C. X-ray diffraction (XRD) was used to determine the bulk structure, and transmission electron microscopy (TEM) was used to determine the microstructural details.

## 2. Experimental

Sample preparation will be described in three parts: electrodeposition of the Co-W alloy, preparation of XRD specimens, and preparations of TEM specimens. The graphite fibers used in this study arrived from the manufacturer packaged in a bundle (tow) of 3000 fibers. The fibers were Hercules AS4-3K fibers^2^ (i.e., polyacrylonitrile-based (PAN) graphite fibers) with a diameter of 7 μm. A 5-cm length of the tow was held stationary in a glass holder during the electrodeposition process. The initial step of the process was a chemical cleaning of the fibers in freshly prepared sulfuric acid solution having a concentration of 0.6 mol/L for 30 s, followed by a distilled water rinse. The Co-W alloy coating was electrodeposited immediately using the electrolyte and conditions given in Table 1. These conditions were shown in a previous communication to produce a smooth coating with the least internal strain associated with hydrogen absorption during the deposition process [3].

The electrodeposited fibers were divided into three portions, and an XRD specimen was prepared from one of the portions of the as-deposited coated fibers. A glass slide was used for the mounting support, with the fibers being laid parallel on doublestick tape and pressed gently to ensure contact with the tape. This mounting technique is commonly used for certain types of powder diffraction specimens, and it is reported that reliable results can be obtained from such a specimen if the operator takes into account the possibility that the specimen may not be infinitely thick [5,6]. The second and third portions of the as-deposited fibers were annealed at 1100 °C for 1.5 h in a sealed quartz tube evacuated to 10^−5^ Pa. The annealed fibers from the second portion were then laid on double-stick tape on a glass slide in the manner already described for XRD specimen preparation. The Co-W coating was removed from the third portion by soaking in 0.6 mol/L H_2_SO_4_ (i.e., 20 % by volume), followed by rinsing with distilled water. These “acid-stripped” fibers were then mounted for XRD analysis in the same manner. Care was taken in positioning the fibers on the focusing circle of the XRD instrument. This was accomplished by shimming the glass slide mount so that the specimen was positioned correctly in the diffractometer within ± 4 μm.^3^ XRD analyses were obtained using a diffractometer equipped with a graphite-diffracted beam monochrometer. Cu *K*α radiation was used to analyze the fiber specimens over a 2*θ* range of 15° to 154° (measured with respect to the fiber axis) with a sampling step of 0.02° and a dwell time per point of 2.5 s. The dwell time per point was increased to 20 s in order to obtain a better signal-to-noise ratio for lattice parameter determination. A calibration curve for the diffractometer was determined using NIST standard reference material SRM 660 [7]. This external correction was then applied to each experimental spectrum to correct for systematic error.

Two types of TEM specimens were prepared from the annealed Co-W coated graphite fibers. The first type was prepared by pulverizing the annealed coated fibers between two sapphire surfaces and placing an acetone suspension of this powder on a carbon grid. The second type provided a cross-sectional view of the coated fibers. Preparation of this type of specimen was accomplished as follows: First, metallic silver was introduced into the annealed coated fibers to serve as an internal lattice-parameter standard for the TEM analysis. This was accomplished by touching a tiny drop of colloidal silver to the tips of the annealed coated fibers, which resulted in capillary action carrying the silver suspension along the regions between adjacent coated fibers and also into hollow regions where the coating and fiber were not perfectly attached. Then a nickel matrix was electroformed around the fibers from a nickel sulfamate electrolyte [8]. The resulting cylindrical metal matrix composite was cross-sectioned with a diamond wafering blade. Disks of 3 mm diameter were spark-cut from the 250 μm thick slices, thinned to about 70 μm by grinding with SiC paper, and polished with an aqueous 1 μm diamond suspension. The polished disks were dimpled on one side to about 15 μm to 20 μm in thickness using diamond paste, polished with 0.05 μm alumina suspension, and ion-milled with an argon beam of 4° incidence. Conventional TEM observations were carried out using Philips 120 kV and 300 kV microscopes, and high resolution TEM was performed using a 400 kV microscope.

## 3. Results and Discussion

### 3.1 SEM/EDS and XRD Analyses

Elemental composition of the Co-W coatings was determined by scanning electron microscopy with energy dispersive x-ray spectroscopy (SEM/EDS). Analyses of polished cross-sections of the coated fibers showed that the mole fraction of tungsten contained in the alloy was (7.10 ± 0.82) % in the as-deposited condition and (5.30 ± 0.66) % after annealing at 1100 °C for 1.5 h.^4^ The fibers and coatings were also analyzed by XRD from specimens that were prepared as described in the experimental section above.

**Fig. 1:**
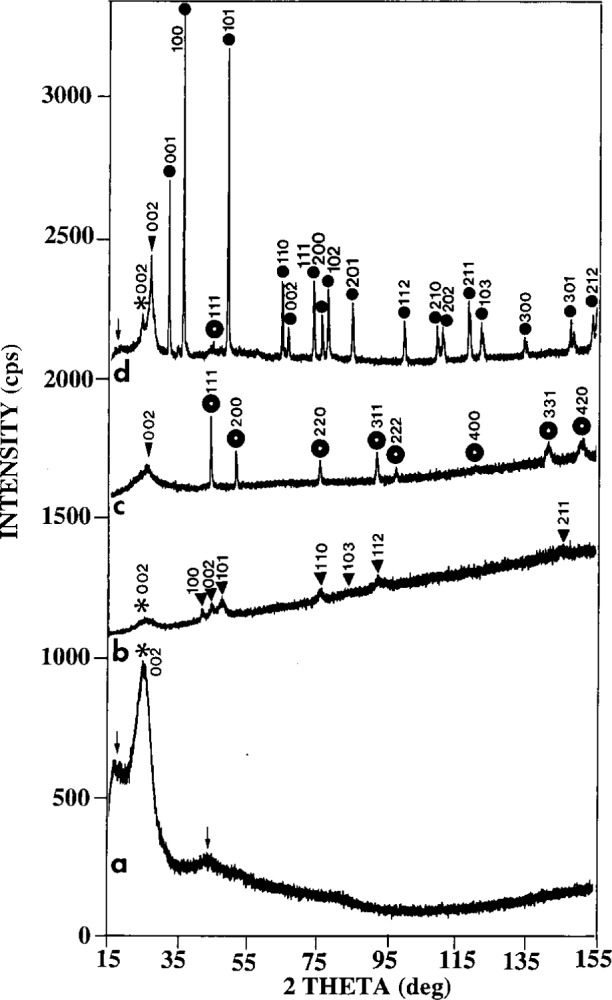
XRD spectra for (a) as-received PAN graphite fibers, (b) Co-W coated fibers (unannealed), (c) Co-W coated fibers (annealed), and (d) fibers after annealing and acid stripping. The peaks are indicated by: ↓ for double-stick tape; ✽ for turbostratic graphite; ∇ for normal graphite; ▼. for h.c.p. Co-W alloy; ✫ for f.c.c. Co-W alloy; and ● for WC. (The spectra are offset for clarity.)

A comparison of the XRD spectra for the PAN graphite fibers during the following stages: as-received, coated, annealed, and acid-stripped, is shown in Figs. 1a–1d. The apparent shoulder at approximately 17° in Fig. 1a is due to the convoluted spectra of the double-stick tape and the glass slide mount. This amorphous glass/tape hump is also slightly visible in Fig. 1d. The fact that the fiber spectra are superimposed on the spectra of the mounting media in Figs. 1a and 1d but not the coated fibers in Figs. 1b and 1c indicates that the x-ray beam is completely attenuated by the coated fibers but not by the uncoated fibers.

In discussing the XRD spectra, we shall first compare the 002 graphite reflection of the unannealed fibers (Fig. 1a) with that of the bare fiber after annealing at 1000 °C for 1.5 h and acid stripping (Fig. 1d). The purpose of this comparison is to identify the changes that occurred in the fiber structure during the annealing process. Although the intensities of the 002 reflections are affected by their superposition with the broad amorphous hump of the glass/tape spectrum, the positions and relative widths of the peaks are not affected. Table 2 presents the 2*θ* and corresponding *d*-spacings for the (002) planes of the as-received and the acid-stripped specimens (Figs. 1a and 1d, respectively). The as-received specimen had a *d*_002_ value of (0.3541 ± 0.0002) nm, which is between the literature values reported for carbonized and graphitized fibers (i.e., 0.361 nm and 0.342 nm respectively) [9].^5^ The peak is quite broad (i.e., the full-width-half-maximum value (FWHM) is 6.66° ± 0.12°), indicating very small crystallites. This is consistent with the fibers being composed of turbostratic graphite [9].

**Fig. 2:**
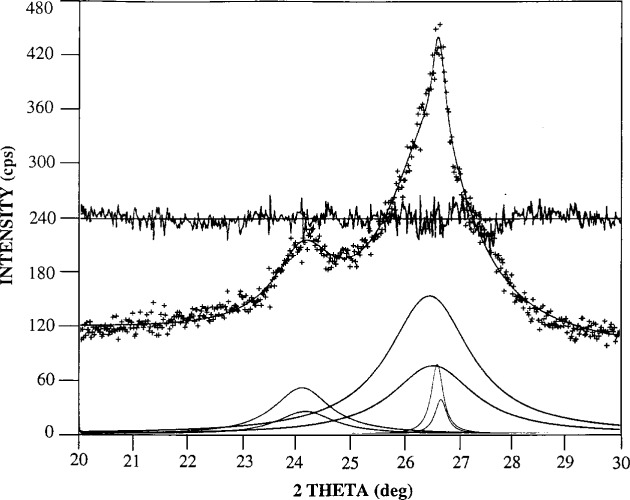
Profile of 002 graphite peak from XRD spectrum of graphite fibers after annealing at 1100 °C for 1.5 h in the presence of Co-W alloy and then acid stripping to remove the alloy coating.

Analysis of the 002 peak of the annealed-fiber spectrum (Fig. 1d) reveals that changes have occurred in the graphite. Profile fitting (Fig. 2) shows that there are three peaks present in the 002 region, corresponding to *d*-spacings of: (0.3700 ± 0.0006) nm; (0.3375 ± 0.0002) nm; and (0.3360 ± 0.0002) nm.^6^ The first of these three values represents a 0.0159 nm increase in *d*_002_, for which there is no obvious explanation. The FWHM of this peak is 1.14° ± 0.14°, much narrower than that of the unannealed graphite, indicating that the crystallite size of the turbostratic graphite has increased significantly upon annealing. The second and third *d*_002_ values are consistent with normal graphite [10]. The FWHM values for these two peaks indicate a bimodal size distribution of the crystallites. The second peak is approximately five times wider than the third peak and three times narrower than the original turbostratic peak. Thus, the second peak seems to represent a transition between the tiny turbostratic graphite crystallites and the large normal graphite crystals. It is apparent from these spectra that the fibers were initially composed of small crystallites of a turbostratic nature. Some turbostratic character remains after annealing, although a significant amount of the graphite has apparently restructured to form larger crystals having the smaller interplanar spacing characteristics of normal graphite. These findings are consistent with literature reports of structural changes occurring in graphite fibers due to dissolution and subsequent recrystallization of the graphite as cobalt or nickel diffuse into the fibers [9].

The remaining peaks in the spectrum in Fig. 1d are consistent with WC, and all of the peaks are accounted for by the reference pattern [10]. As already mentioned, SEM/EDS analysis showed the tungsten composition of the alloy to have decreased significantly upon annealing. It is apparent from the acid-stripped XRD spectrum (Fig. 1d) that some of that missing tungsten went into forming WC at the coating/fiber interface. The presence of WC points to a solid state reaction between tungsten and carbon. This evidence indicates that carbon and tungsten interdiffused during the annealing process. The graphite recrystallization evidence indicates that cobalt also diffused into the fibers.

Figs. 1b and 1c are the spectra of the Co-W coated fibers before and after annealing. All of the non-graphite peaks in the Fig. 1b match hexagonal close-packed (h.c.p.) Co, and their breadth indicates that the crystallites are very small. This finding is consistent with reports from other researchers concerning Co-W alloys that were electrodeposited from acidic cobalt-tungsten electrolytes [11,12]. Fig. 1c is a spectrum of the alloy-coated fibers after annealing at 1100 °C for 1.5 h. The non-graphite peaks in the spectrum correspond to face-centered cubic (f.c.c.) cobalt whose peaks are shifted to slightly lower angles. The evidence for lattice expansion will be discussed in detail after a brief description of a relevant study from the literature.

Roebuck, Almond, and Cottenden [13] investigated the effect of various tungsten and carbon compositions on the structure and mechanical properties of Co-W-C alloys. They found that the extruded alloys with mole fraction of tungsten of 5.9 % were entirely f.c.c., provided that their mole fraction of carbon was at least 0.1 %.^7^ Alloys containing less tungsten required more carbon. For example, alloys with a mole fraction of tungsten of 2.7 % tungsten required the mole fraction of carbon to be 0.3 % in order to stabilize the f.c.c. phase [13]. Since our annealed specimens appear to be entirely f.c.c. with a mole fraction of tungsten of (5.30 ± 0.66) %, it follows that they probably also contain carbon. Since a direct method for accurately measuring the carbon composition of this alloy in the presence of the graphite fibers was not available, an alternative indirect method was employed to estimate the concentration. This method uses an empirical equation to calculate the amount of carbon in the Co-W-C alloy of interest. The equation, *a*^fcc^ = *a*^fcc^_0_ + 0.00036*m*_W_ + 0.0012*m*_C_ (which was reported by Roebuck, Almond and Cottendon [13]) will subsequently be referred to here as the RAC equation. The parameters needed to perform the calculation are: *a*^fcc^, the lattice parameter of the Co-W-C alloy; *a*^fcc^_0_, the lattice parameter of f.c.c. cobalt (0.35447 nm) [10];^8^ *m*_W_, the mole fraction of tungsten (%); and *m*_C_, the mole fraction of carbon (%) present in the alloy. Thus, before we can calculate *m*_C_, it is necessary to first determine the lattice parameter of the alloy.

The usual method for precise determination of the lattice parameter of a crystalline phase involves plotting the nominal lattice parameter (calculated from the *K*α_1_ peak position) versus sin^2^*θ*. Greatest accuracy is obtained by using only those peaks for which *θ* ≥ 60°, thus minimizing the error in sin^2^*θ* [14]. The only high-angle cobalt peaks which are accessible by Cu *K*α radiation are 400, 331, and 420. Of those three, only the 331 peak was found to be free from interference by either WC or the pyrolytic graphite. Thus the 331 peak alone was selected for the lattice parameter determination in order to be sure that only the reflections from the Co-W alloy were considered. Using only one peak, however, precluded the precise determination of the lattice parameter, since that method requires graphical extrapolation involving several reflections. The error in sin^2^*θ* can be considered minimal, however, since the 331 reflection occurs at such a high angle. Hence, although the values determined here are not precise lattice parameters, they can be considered to be good approximations.

**Fig. 3:**
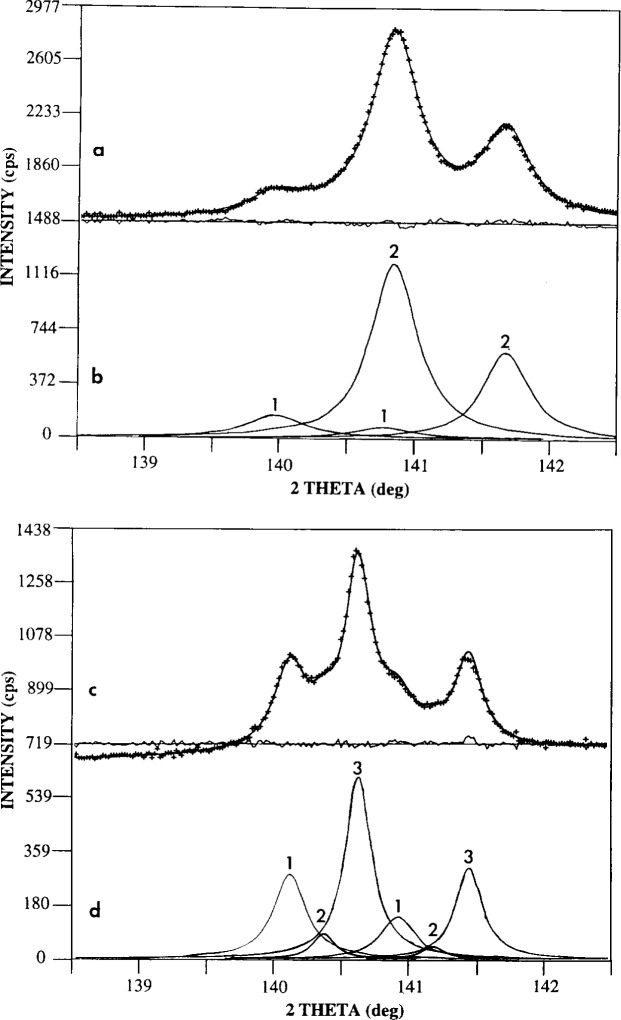
XRD spectra of Co-W alloy on (a) flat pyrolytic and (c) fiber turbostratic graphite substrates after annealing at 1100 °C for 1.5 h. Peak profiles of (a) and (c) are presented in (b) and (d), respectively.

XRD spectra for the lattice parameter determination were run with 20 s dwell time per point. Close inspection of the individual peaks showed that each was really a convolution of more than one *K*α_1_–*K*α_2_ pair. Three tests were applied to determine whether the multiple pairs of peaks might actually be artifacts resulting either from variations in position among the fibers with respect to the x-ray beam or from the unusual geometry of the specimen (i.e., parallel fibers instead of powder). First, a calculation was made to determine the angular difference for the arrival of the diffracted beam at the XRD detector for two coated fibers if one of the fibers was correctly positioned and the other was located just beneath it. This calculation showed that the resulting difference in 2*θ* at the detector would be 0.003° for a coated fiber with a diameter of 20 μm. This value is much smaller than the angular difference between the convoluted experimental peaks (i.e., approximately 0.5° in the case of the 331 peak); and hence it is not a satisfactory explanation of the observed multiple peaks. The second test involved running a spectrum of another specimen with a similar geometry (i.e., nickel-coated graphite fibers) to see if more than one *K*α_1_–*K*α_2_ pair was observed for each of the f.c.c. nickel reflections. If the presence of multiple peaks in the Co-W alloy were really a geometrical artifact, then the nickel spectrum should also have contained convoluted peaks. The fact that it did not indicates that the multiple peaks are characteristic of the annealed Co-W/graphite spectrum, and they arise from the crystallographic nature of that alloy. A third test was then applied: the alloy was deposited on a different type of graphite substrate, and the experiment was repeated. Flat pyrolytic graphite was used as the substrate, and the as-coated sample was annealed in the same manner as the coated fibers. The resulting XRD spectrum on the coated flat specimen (Fig. 3a) also contained multiple peaks. However, the flat specimen (Figs. 3a and 3b) has two *K*α_1_–*K*α_2_ pairs, and the fiber specimen (Figs. 3c and 3d) has three. This may be due to the difference in the initial character of the graphite substrates, since the flat was pyrolytic and the fiber was turbostratic. Pyrolytic graphite has a more perfect lattice than turbostratic graphite. Hence, individual carbon atoms from the pyrolytic lattice would be more resistant to dissolution in the Co-W alloy than those from the turbostratic [9]. Thus, the amount of carbon dissolved in the alloy would be expected to be somewhat lower for the flat samples after annealing for an equal amount of time. Assuming that each *K*α_1_–*K*α_2_ pair for a given reflection (e.g., 331 reflection) would correspond to a slightly different lattice parameter, a determination of the lattice parameters might provide important information regarding the coating.

The spectra for the 331 reflections of the flat and fiber specimens are shown in Figs. 3a and 3c, and their respective peak profiles are shown in Figs. 3b and 3d. Table 3 lists the 2*θ* as well as the *d*_331_ and lattice-parameter values corresponding to the deconvoluted peaks. The two sets of peaks from the flat sample appear to correspond to the first and third of the fiber sample peaks. However, the second (smallest) of the fiber sample peaks has no apparent counterpart in the flat sample. This will be discussed later.

**Fig. 4:**
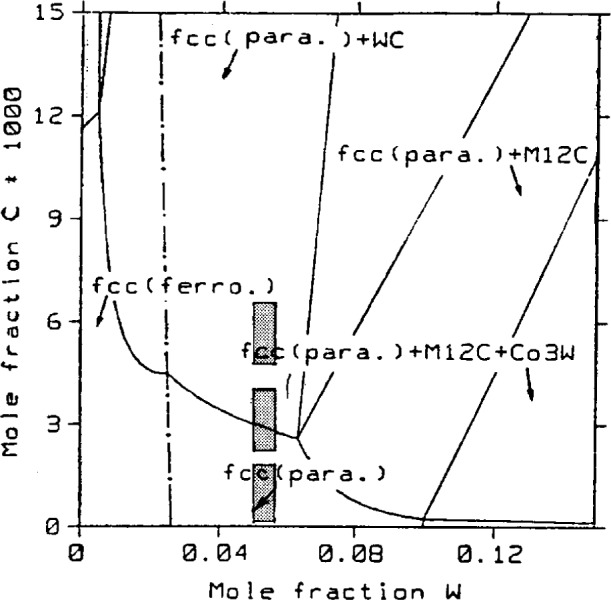
The Co-rich corner of the Co-W-C phase diagram at 1000 °C, according to Guillermet [15]. The three W-C compositions indicated by shading correspond to the three sets of 331 *K*α_1_-*K*α_2_ pairs in the XRD spectrum of Fig. 3d. (The concentration ranges indicated for tungsten and carbon represent the expanded uncertainties *U* = *ku*_c_ for the tungsten and carbon compositions, respectively. They were determined from the respective combined standard uncertainties *u*_c_ for the two elements and a coverage factor *k* = 1. The unknown values can, therefore, be asserted to lie within the shaded regions defined by *U* with a confidence of 68 %.)

We are now in a position to apply the RAC equation, having already determined the tungsten composition and the approximate lattice parameter of the alloy. The carbon compositions, which were determined by applying the equation to the three 331 *K*α_1_ peaks of the coated fibers, are given in Table 3. It is interesting to compare this experimental f.c.c. Co-W-C alloy with the Co-rich corner of the 1000 °C Co-W-C phase diagram of Guillermet [15]. Figure 4 shows the three regions corresponding to the three calculated carbon compositions of the alloy superimposed upon Guillermet’s diagram.^9^ It appears that the first and third XRD peaks correspond to two separate areas of the phase diagram, and the second peak corresponds to the boundary between the single-phase and the two-phase regions. Thus, at equilibrium, the lower carbon content (higher angle peak) corresponds to a single-phase paramagnetic f.c.c. region, and the higher carbon (lower angle peak) corresponds to a two-phase paramagnetic f.c.c. region (i.e., f.c.c. + WC). The fact that the lattice parameter continues to increase for carbon compositions corresponding to the two phase region, however, indicates that the alloy is supersaturated and not at equilibrium. The diagram indicates that under equilibrium conditions the lattice parameter of the f.c.c. alloy in the two phase region would remain constant with increasing carbon concentration, and the additional carbon would be precipitated as WC. Consequently, one would expect that the lattice parameter would actually decrease as the excess carbon depleted the tungsten concentration. Hence, the relative percentage of WC would increase with respect to the f.c.c. phase. Peaks corresponding to carbon compositions in both the single-phase and two-phase regions are also observed in the spectrum of the flat graphite. But, the low carbon phase dominates there, as indicated by the relatively high intensity of the corresponding reflection. This observation is consistent with a lower carbon dissolution rate, as would be expected for the pyrolytic graphite.

### 3.2 Microstructural Determination by TEM

**Fig. 5:**
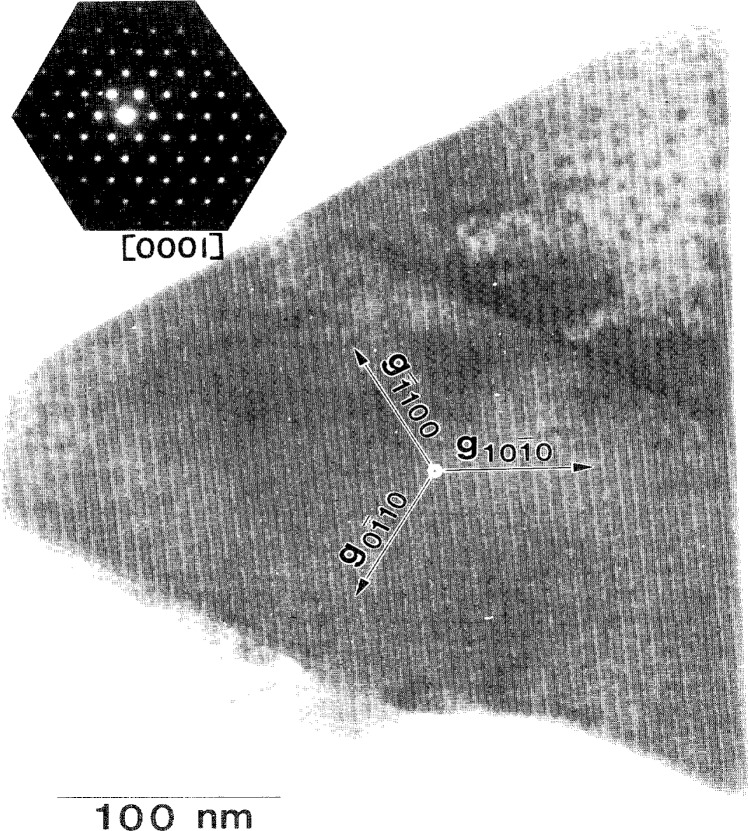
Triangularly faceted crystal of WC, found in powdered specimen prepared from Co-W-coated graphite fiber after annealing at 1100 °C for 1.5 h.

TEM analyses were then performed to determine the local microstructures. Initial TEM inspection was carried out using specimens prepared by crushing the 1100 °C-annealed Co-W-coated fibers. Numerous triangularly-faceted crystals, such as the one shown in Fig. 5, were observed by TEM and analyzed by EDS. The diffraction patterns from the former and the spectra from the latter were consistent with hexagonal tungsten carbide, WC. The question of how the WC, the fiber, and the cobalt alloy were related could only be answered by a cross-sectional view of the coating-fiber interface.

**Fig. 6:**
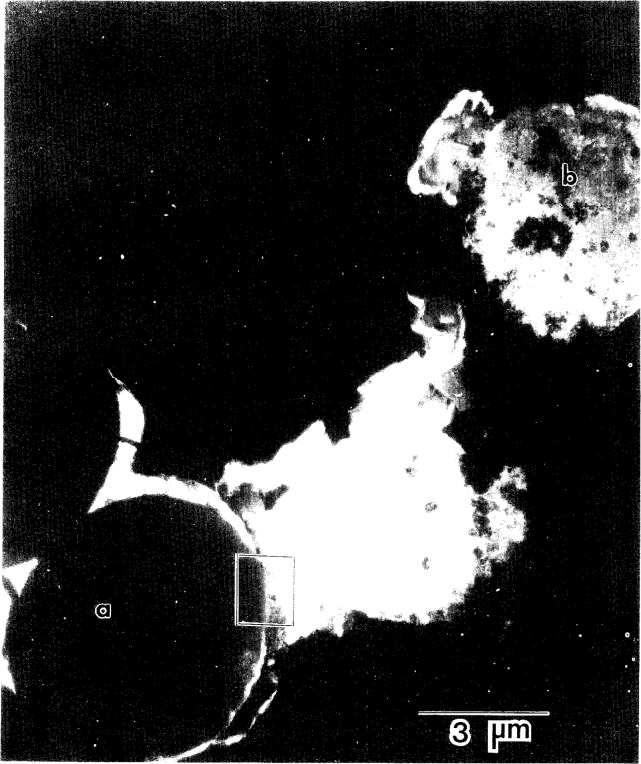
Transverse cross-sectional view of alloy-coated graphite fibers after annealing at 1100 °C for 1.5 h and electroforming into a nickel matrix composite. Fiber **a** debonded before or during annealing, whereas fiber **b** remained sufficiently well-bonded that it underwent diffusion-induced reactions.

**Fig. 7:**
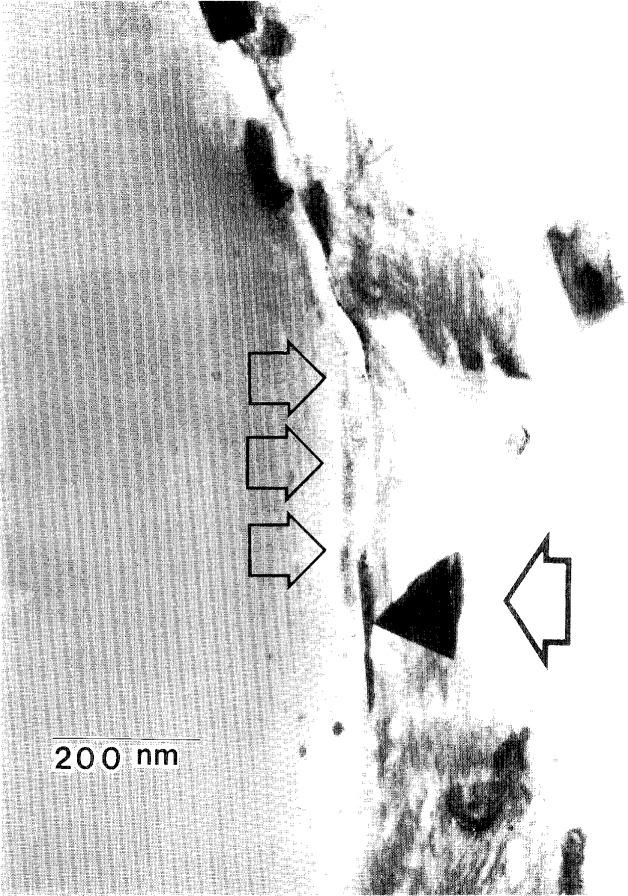
Higher magnification micrograph of the boxed region in Fig. 6. The single arrow points to the triangularly faceted WC crystal in the interface of the poorly bonded coating-fiber interface, while the set of three arrows indicates the region in which other crystals are lying flat with respect to the interface.

Figure 6 shows such a cross-sectional view. Although the Co-W-coated graphite fibers were initially virtually identical, microstructural variations were apparent after annealing for 1.5 h in vacuum at 1100 °C. For example, the cross-section of fiber **a** closely resembles an unannealed fiber in size and shape, whereas fiber **b** is reduced in size and has an inhomogeneous appearance with an irregular border. It is apparent that the “**b**”-type fibers underwent significant diffusion-induced reaction during annealing. The coating/fiber interfacial region indicated by the box in Fig. 6 is shown at a higher magnification in Fig. 7, revealing the presence of a triangularly faceted crystal which is indicated by a single arrow. The other three arrows point to flat crystals, which are also in the interfacial region. Although it is not clear whether they are really thin triangular crystals lying flat against the fiber, it seems likely since such an orientation would be perpendicular to both the carbon and tungsten diffusion fluxes.

**Fig. 8:**
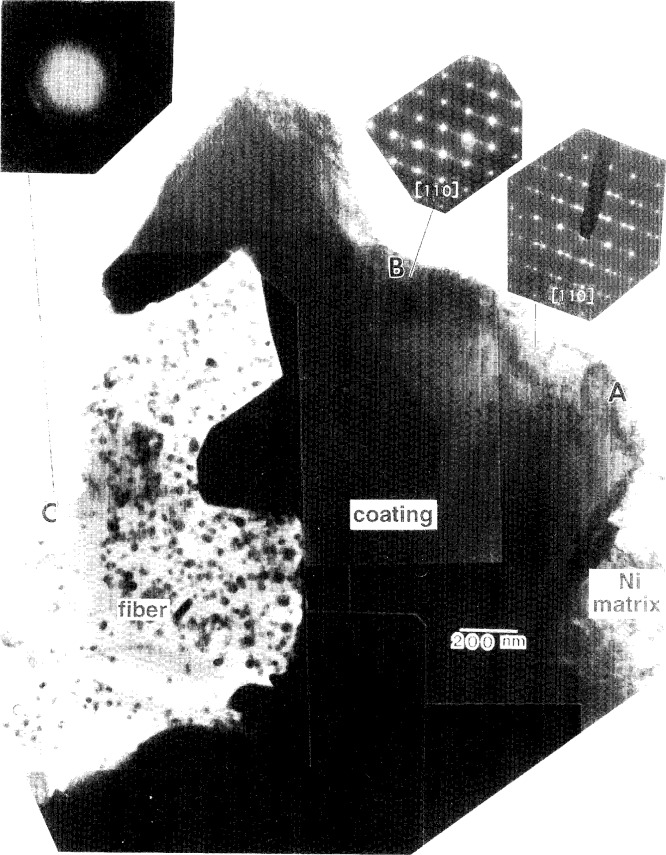
Composite micrograph showing a portion of another well-bonded coating-fiber interface. SAD patterns of two areas of the coating (regions **A** and **B**) and the reacted fiber (region **C**) are inset.

The coating-fiber interface observed in the composite micrograph in Fig. 8 shows ovoid-shaped nanocrystals of silver within the fiber itself. They are there as a result of the wicking of the colloidal silver through channels that were formed throughout the fiber during the annealing process. Thus the silver internal standard was incorporated into the observation plane where it could be used for lattice spacing determination, as will be discussed shortly. The obviously porous nature of the fiber and the faceting of the surrounding coating are consistent with a coating-fiber interface that underwent extensive diffusion of carbon out of the fiber as cobalt and tungsten diffused into the fiber. The irregularity of the coating boundary indicates that the rate of diffusion was not constant around the fiber perimeter. This may be attributed to localized diffusion conditions resulting from the crystallographic orientation of the different Co-W grains touching the fibers and to variations in the microstructure of the fiber itself.

**Fig. 9:**
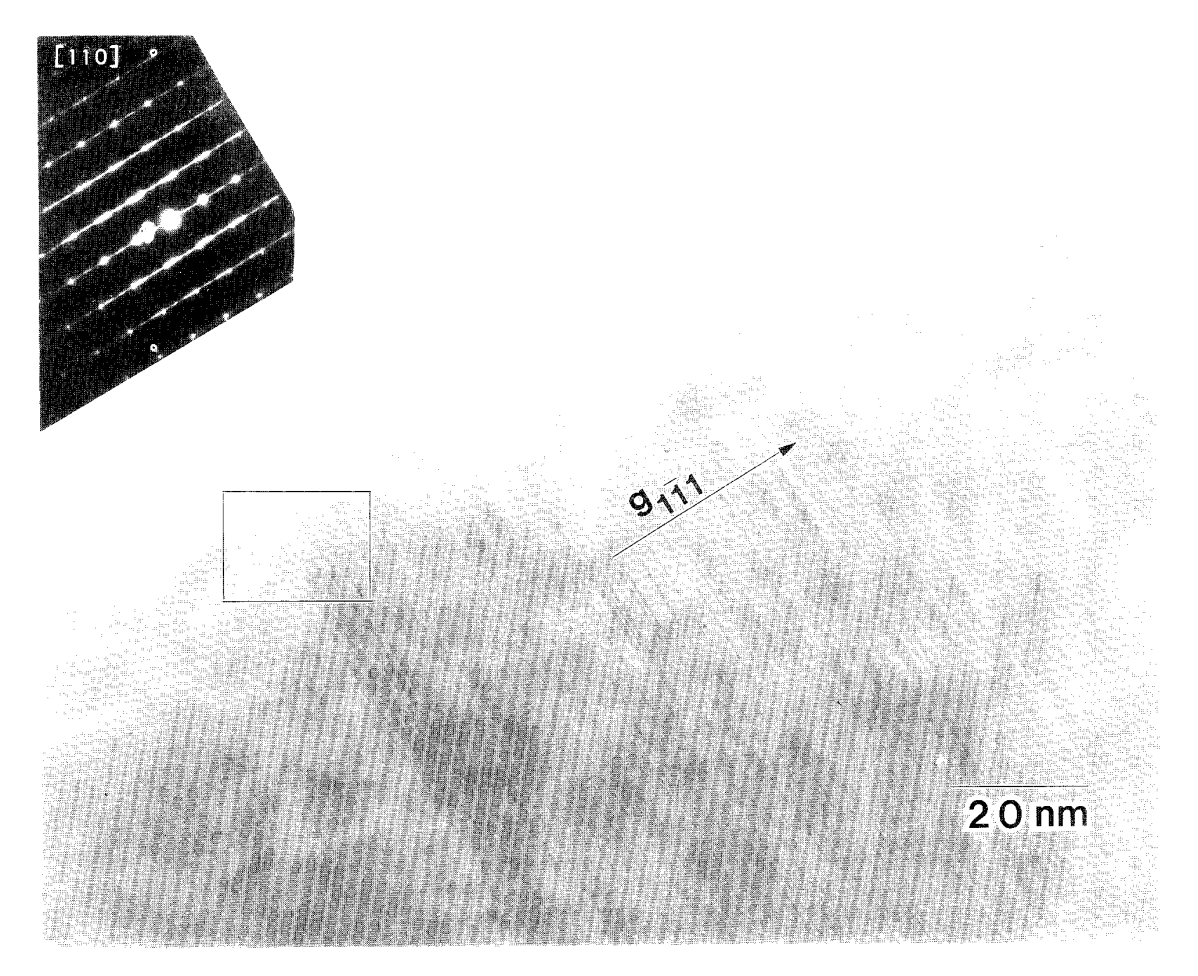
Micrograph of region **A** of coating from Fig. 8. The inset SAD pattern shows streaking in the $\left[1\bar{1}\bar{1}\right]$ direction, which conforms to the striated appearance of the corresponding planes of the micrograph; twin spots are also present.

**Fig. 10:**
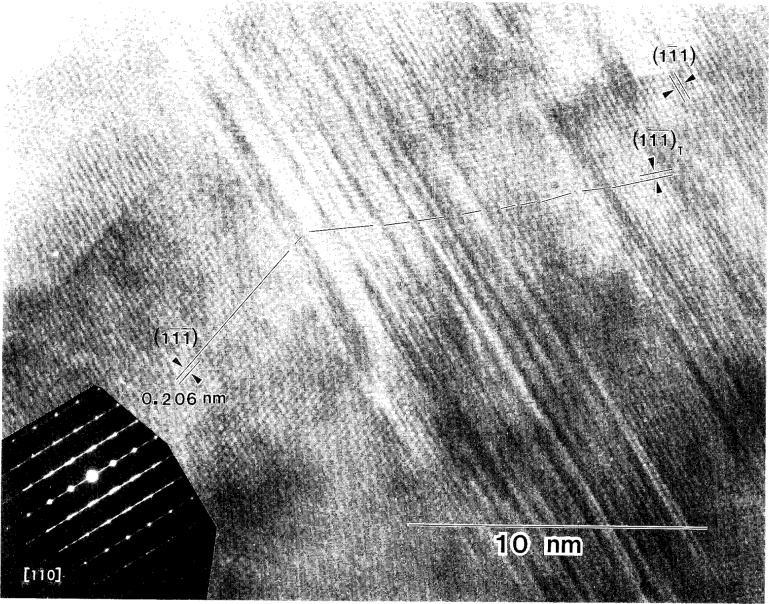
High-resolution micrograph of region **A** from Fig. 8 (zone axis [110]). Region containing stacking faults forms a transition between f.c.c. annealing twins. Lattice fringes for the $\left(1\bar{1}\bar{1}\right)$, ${\left(1\bar{1}\bar{1}\right)}_{T}$ (i.e., twin) and $\left(1\bar{1}1\right)$ planes are indicated.

**Fig. 11:**
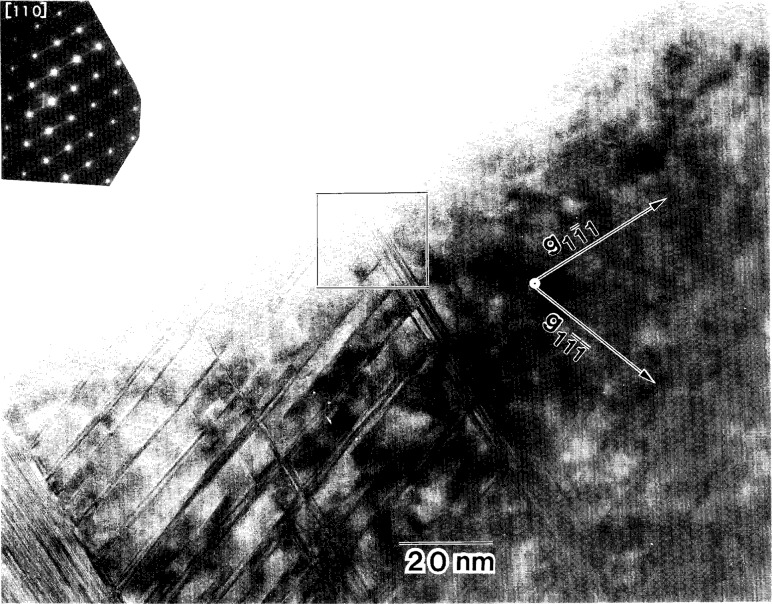
Micrograph of region **B** of coating from Fig. 8. The inset SAD pattern shows streaking in both the $\left[1\bar{1}1\right]$ and $\left[1\bar{1}\bar{1}\right]$ directions, but predominantly the former, corresponding to the stacking faults observed for the corresponding planes in the micrograph.

Two selected area diffraction (SAD) patterns of the coating (regions **A** and **B**) and one of the reacted fiber (region **C**) are inset in Fig. 8. The f.c.c. [110] zone axis pattern for region **A** contains streaking in the $\left[1\bar{1}1\right]$ direction, in agreement with the $\left(1\bar{1}1\right)$ stacking faults which are shown in Fig. 9. The high resolution micrograph of region **A** (Fig. 10) does not, however, show simple reflection twins about the $\left(1\bar{1}1\right)$ plane, which would be consistent with the twin spots in the inset SAD pattern and which are common for annealed f.c.c. alloys [14]. Instead, there is a complex arrangement of slightly misaligned $\left(1\bar{1}1\right)$ planes, gradually transitioning to a reflection twin. Stacking faults, comprising the transition region, are responsible for the streaking. The enlargement of region **B** (Fig. 11) contains stacking faults on both the $\left(1\bar{1}1\right)$ and $\left(1\bar{1}\bar{1}\right)$ planes, in agreement with the inset SAD pattern, which shows streaking in the $\left[1\bar{1}1\right]$ and $\left[1\bar{1}\bar{1}\right]$ directions. It has been shown that high concentrations of hydrogen can result in high densities of stacking faults in cobalt alloys [16]. Although the hydrogen concentration was not measured, this does not seem likely to be the cause of the stacking faults in the present case. If it were, the stacking fault density would be expected to be rather consistent throughout the alloy, since there would be no apparent reason for the hydrogen concentration to be inhomogeneous. In fact, however, the stacking fault density is very high in some regions and virtually nonexistent in others. Thus, it seems more likely that the stacking fault density in a particular region of the alloy was correlated with the carbon concentration in that region. Since the carbon would have come into the coating by diffusion from the fiber, its concentration would be expected to depend on the location of the region in question with respect to the carbon diffusion gradient in the coating. Assuming that the stacking faults were associated with regions of high carbon concentration caused by carbon diffusion along the {111} planes, then region **A** with a high stacking fault density might correspond to the high carbon phase (i.e., the mole fraction of carbon being 0.66 % ± 0.18 %) that was determined from the XRD data. This hypothesis will be revisited after presentation of some additional electron diffraction data.

There is another feature visible in the SAD pattern of region **A**: a faint ring pattern superimposed upon the f.c.c. cobalt alloy crystal pattern. This ring pattern is consistent with fine crystallites of WC_(1 −_ *_x_*_)_, a high-tem perature f.c.c. tungsten carbide, as shown in Table 4. Since the lattice parameter of the WC_(1 −_ *_x_*_)_ is 0.4248 nm,^10^ the lattice mismatch between this species and the f.c.c. Co-W alloy would be approximately 19 %. Although the microstructural relationship between the alloy and the carbide is not clear, the ring pattern indicates that the carbide crystallites are randomly oriented.

**Fig. 12:**
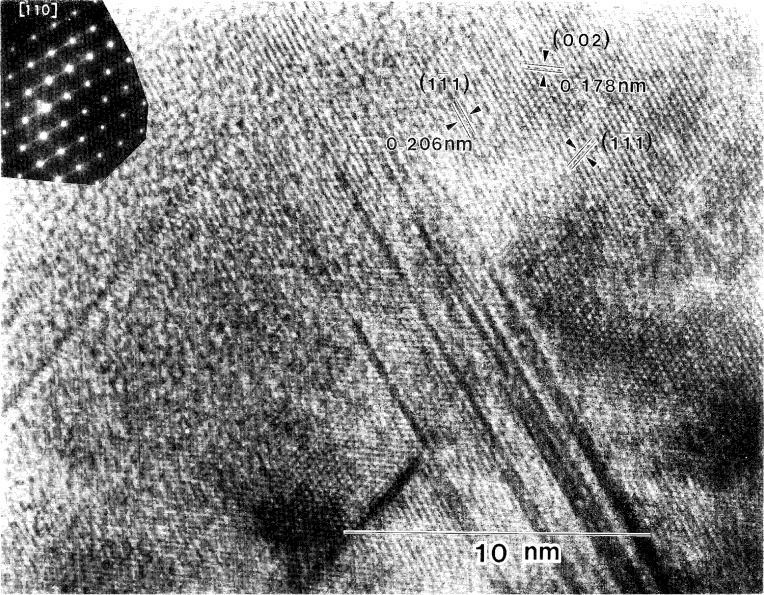
High-resolution micrograph of region **B** from Fig. 8 (zone axis [110]), Lattice fringes for the (002), $\left(1\bar{1}1\right)$, and $\left(1\bar{1}\bar{1}\right)$ planes are indicated.

In contrast to region **A** of Fig. 8 in which the density of stacking faults is very high, region **B** contains 5 to 10 stacking faults grouped closely together (all within approximately 5 nm) on the $\left(1\bar{1}1\right)$ plane with a distance of approximately 50 nm separating one group from the next, as shown in Figs. 11 and 12. Similarly, on the $\left(1\bar{1}\bar{1}\right)$ plane, there are groups of 3 to 5 stacking faults within 5 nm of each other, with each group located approximately 5 nm to 15 nm from the next. If the stacking faults on the {111} planes of the alloy are due to carbon diffusion along those planes, then the pattern in region **B**, which has a lower stacking fault density, presumably indicates a lower carbon concentration than in region **A**. Regions **A** and **B** are both in the same orientation (i.e., both on the [110] zone axis) with no rotation with respect to each other. Thus they evidently are two parts of one grain with variation only in the density and direction of the defects, this being due to variations in carbon concentration. In order to obtain a high enough carbon concentration to achieve the observed stacking fault density in region **A**, another fiber must have been positioned in what is now the perforated region of the sample located just beyond region **A**. With such a geometric relationship between the Co-W grain and the two fibers, carbon would presumably have been able to diffuse into the Co-W grain from both fibers. The facets of the coating at the fiber interface in Fig. 8 correspond to $\left(1\bar{1}\bar{1}\right)$, (220), and $\left(1\bar{1}3\right)$ planes. It is expected that the fiber viewed in three dimensions would have notching all around it and down its length, due to its dissolution and subsequent diffusion through the randomly oriented Co-W grains.

### 3.3 Consideration of the SEM/EDS, XRD, and TEM Evidence

Assuming that the three overlapping 331 peaks in the XRD spectrum of the alloy correspond to regions of different carbon concentration, as discussed earlier, it would be expected that the carbon concentration of the alloy would decrease with increasing distance from the fiber-coating interface. Recall that the TEM/electron diffraction (ED) analysis indicated that regions of high stacking-fault density also contained WC_(1 −_ *_x_*_)_. Supposing *x*->0, then this species of tungsten carbide would differ from WC only by virtue of the former being f.c.c. and the latter being h.c.p. in structure. Although the phase diagram in Fig. 4 indicates the high carbon region would contain paramagnetic f.c.c. alloy + WC, two things must be mentioned here. First, this is an equilibrium phase diagram, and the experimental sample is not in equilibrium. Thus, although the diagram may be helpful in determining what phases are present, it must be considered a general guideline. Second, it is conceivable that the WC might be constrained to be f.c.c. in an effort to be coherent with the alloy. Thus it seems likely that the areas of high stacking-fault density in the TEM analysis of the alloy would correspond to regions of high carbon concentration in the XRD analysis. Since no evidence for a second phase is seen in the SAD pattern of region **B** of Fig. 8, this area may correspond to the low carbon paramagnetic f.c.c. single phase region of the Co-W-C phase diagram. Carrying the association between the HRTEM images and the XRD data a bit further, one might expect that the region containing relatively few stacking faults at the right of Fig. 10 might then correspond to the low-carbon phase (i.e., mole fraction of carbon of (0.19 ± 0.18) %, as reported in Table 3) determined from the XRD.

**Fig. 13:**
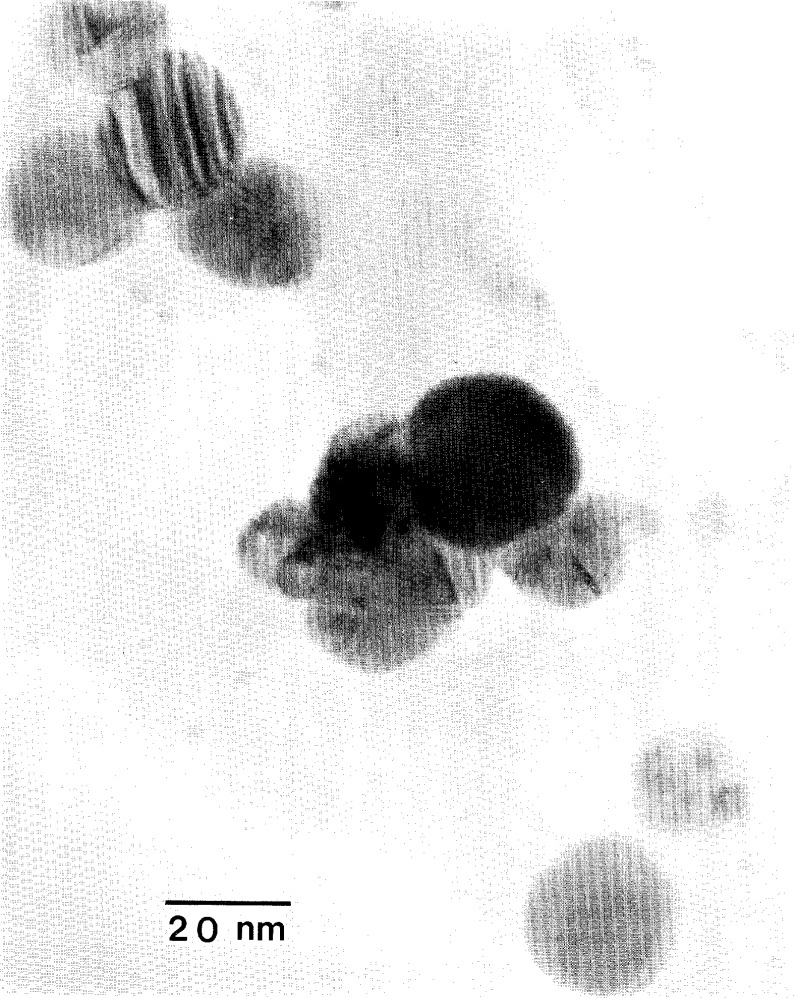
Groups of silver nanocrystals from region **C** of Fig. 8.

**Fig. 14:**
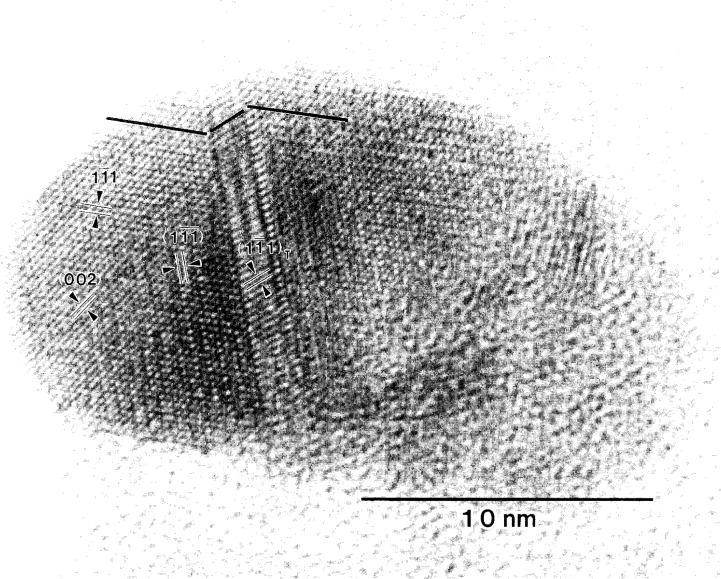
High-resolution micrograph of silver nanocrystal from region **C** of Fig. 8 showing the lattice fringes for the (002), $\left(1\bar{1}1\right)$, $\left(1\bar{1}\bar{1}\right)$, and ${\left(1\bar{1}\bar{1}\right)}_{T}$ (i.e., twin).

Let us now return to region **C** in Fig. 8 and the diffusion-induced changes in the fiber. A closer view of one of the altered fibers (Fig. 13) reveals clusters of spherical to ovoid-shaped silver nanocrystals residing in the pores of what is left of the graphite of the original fiber. A high resolution TEM micrograph of a silver nanocrystal in the [110] zone axis orientation is shown in Fig. 14. Fast fourier transformation (FFT) of the lattice fringes of that crystallite and the $\left(1\bar{1}\bar{1}\right)$ lattice fringes in region **A** of the coating (Fig. 10) permitted a direct comparison. From the silver *d*_111_ spacing (0.2359 nm) [10],^11^ the lattice parameter of the cobalt-tungsten alloy was determined to be (0.357 ± 0.002) nm.^12^ This value agrees with the XRD data. The non-silver Bragg reflections in the SAD pattern of region **C** (inset of Fig. 8) are given in Table 5. Those reflections which are not obscured by the intensity of the center spot and the graphite 002 reflections (0.3348 nm) [10],^13^ agree with WC*_x_*, a hexagonal tungsten carbide species indicating that tungsten must have diffused into the fiber during annealing. Thus the tiny rounded shadowy forms indicated by arrows in the micrograph of Fig. 13 are evidently WC*_x_* nucleii. Judging from the uniformly small size of the crystallites (i.e., approximately 1 nm to 3 nm), nucleation was favored over growth under these annealing conditions.

Summarizing, the XRD data showed the as-deposited Co-W alloy coating to be h.c.p., whereas only the f.c.c. phase was observed after annealing at 1100 °C for 1.5 h. SEM/EDS analysis showed that the as-deposited alloy contained a mole fraction of tungsten of (7.10 ± 0.82) % tungsten, but the annealed alloy contained only (5.30 ± 0.66) %. Evidence of the diffusion of tungsten and carbon and reactions between the two were revealed during the XRD and TEM analyses. Hexagonal tungsten carbide (WC) was observed by XRD on the annealed fiber surface after acid-stripping to remove the coating, indicating that carbon had diffused out and reacted with tungsten in the alloy at the coating-fiber interface. TEM analysis showed WC to be present at some of the fiber interfaces in the form of flat triangular crystals, having sides approximately 100 nm to 300 nm in length. The WC crystals seemed to form an overlapping network, lying flat against the fiber surface, which could explain why the coating increases the lifetime of the fibers but does not protect them completely. Those fibers that appeared unchanged by the annealing process had this type of protective network of overlapping WC crystals. However, the fibers having no apparent WC crystals at the interface had irregular borders arising from diffusion of the carbon out of the fiber and into the alloy. TEM/SAD analysis showed such fibers to contain nanocrystals of WC_1 −_ *_x_*. The shift in the graphite 002 reflection in the XRD spectrum revealed that annealing had almost entirely converted what was initially turbostratic graphite to normal graphite with smaller *d*_002_ spacing. Although the XRD spectrum of the annealed coated fibers indicated that some of the graphite still had the wide turbostratic *d*_002_ spacing, the intensity of the *d*_002_ peak corresponding to normal graphite was much greater, indicating that the majority of the fibers had been altered by the annealing process. Thus, while it is not clear why the WC network formed around some fibers and not others, it can be concluded that the presence of this network is responsible for the success of the Co-W alloy coating as a diffusion barrier and the absence of the network for the failure of the coating at 1100 °C.

Inspection of the XRD peaks of the annealed f.c.c. alloy showed each peak to be convolutions of multiple peaks. Deconvolution of the 331 peak, followed by lattice parameter determination from the deconvoluted peaks, indicated that three distinct f.c.c. Co-W-C alloy compositions may have formed upon annealing the coated graphite fibers at 1100 °C for 1.5 h. It appears that small differences in carbon composition, ranging from a mole fraction of carbon of 0.19 % to 0.66 % (± 0.18 at. %), are responsible for the change in lattice parameter. It is suggested that: (1) areas with very high stacking fault densities such as region **A** (shown in the TEM micrograph in Fig. 8 and in higher magnification in Figs. 9 and 10) have the highest carbon concentration; (2) the medium carbon concentration corresponds to areas such as region **B** (shown in Fig. 8 and in higher magnification in Figs. 11 and 12); and (3) the low carbon concentration corresponds to the relatively un-faulted region shown on the right in Fig. 11 and in those regions surrounding fibers that were protected by a network of overlapping WC crystals.

## Figures and Tables

**Table 1:** Preparation and use of Co-W electrolyte used to produce Co-W alloy diffusion barrier

0.23 mol/L cobalt sulfate
0.057 mol/L sodium tungstate
0.31 mol/L citric acid
pH 3 (adjusted with ammonium hydroxide)
Use at room temperature with a current density of 30 mA/cm^2^

**Table 2:** 002 graphite peaks in XRD spectra of graphite fibers in the as-received state and after annealing in vacuum for 1.5 h at 1100 °C in the presence of Co-W alloy. (Note: The alloy coating was removed by acid etching before running the spectrum)

Treatment	2*θ* (degrees)^a^	FWHM (degrees)^a^	*d* (nm)^b^	Type
Unannealed	25.13±0.01	6.66±0.06	0.3541±0.0002	turbostratic
Annealed	24.04±0.02	1.14±0.07	0.3700±0.0006	turbostratic
	26.39±0.01	2.19±0.06	0.3375±0.0002	normal
	26.51±0.01	0.42±0.03	0.3360±0.0002	normal

a The quoted uncertainty is an expanded uncertainty *U* = 2*u*_c_, where the combined standard uncertainty *u*_c_ is the standard deviation calculated by the Diffrac 5000 program [7].

b The values of *d* and the quoted expanded uncertainties *U* = 2*u*_c_ reported here were calculated from the 2*θ* values.

**Table 3:** Deconvolution of 331 peaks in XRD spectra of Co-W alloy that was electrodeposited onto pyrolytic graphite (flat) and turbostratic graphite (fiber) substrates and annealed at 1100 °C for 1.5 h

Substrate	2*θ* (degrees)	*d*_331_ (nm)	*a* (nm)	*m*_C_ (%)
pyrolytic (flat)	140.08^a^	0.08195	0.35722	
	140.96^a^	0.08173	0.35623	
turbostratic (fiber)	140.22±0.14^b^	0.08191±0.00004^c^	0.35706±0.00004^c^	0.66±0.36^d^
	140.49±0.18^b^	0.08184±0.00004^c^	0.35675±0.00004^c^	0.40±0.36^d^
	140.72±0.08^b^	0.08179±0.00002^c^	0.35650±0.00002^c^	0.19±0.36^d^

a Result of a single measurement.

b Mean and expanded uncertainty (*U* = 2*u*_c_) values from six spectra.

c Values calculated from the mean and expanded uncertainty values (*U* = 2*u*_c_) in 2*θ*.

d Mean and expanded uncertainty (*U* = 2*u*_c_) in the carbon composition.

**Table 4:** Comparison of SAD ring pattern from region **A** of Fig. 8 with JCPDS diffraction pattern for WC_1 −_ *_x_*_)_ [16]

Experimentally determined *d* (nm)	JCPDS value of *d* for [WC_(1 −_ *_x_*_)_] (nm)	*hkl*	Ratio *d* (exper.)/*d* (JCPDS)
0.243±0.008^a^	0.2429	111	1.00
0.212±0.008^a^	0.2107	200	1.01
0.149±0.008^a^	0.1495	220	1.00
0.127±0.008^a^	0.1277	311	1.00
0.121±0.008^a^	0.1221	222	0.99
0.107±0.008^a^	0.1061	400	1.01
0.094±0.008^a^	0.0973	331	0.97
0.094±0.008^a^	0.0948	420	0.99

a The expanded uncertainty *U* = 2*u*_c_ in the measured *d*-spacings was calculated from the standard deviations in the camera constant and in the measurement of the diameters of the experimental rings. The mean and the standard deviation of the camera constant were determined from five measurements each of the diameters of the first seven rings in a SAD pattern from a microcrystalline aluminum standard. The mean and standard deviation of the SAD rings of the experimental sample were determined from three to five measurements of the diameters.

**Table 5:** Comparison of SAD ring pattern from region **C** of Fig. 8 with JCPDS pattern [16]^a^

Experimentally determined *d* (nm)	JCPDS value of *d* for Wc*_x_* (nm)	*hkl*	Ratio *d* (exper.)/*d* (JCPDS)
b	0.461	200	
b	0.415	112	
b	0.401	103	
b	0.348	210	
0.246±0.004^c^	0.2455	222	1.00
0.228±0.004^c^	0.2283	400	1.00
0.197±0.004^c^	0.2001	410	0.98
0.144±0.004^c^	0.1416	208	1.02
0.133±0.004^c^	0.1309	700;350	1.02
0.124±0.004^c^	0.1228	256	1.01

a Those reflections corresponding to silver (the high-resolution TEM lattice standard) are excluded; but all others are reported in this table.

b It was not possible to observe reflections with indices lower than 222, since they were obscured by the graphite 002 reflections.

c The expanded uncertainty *U* = 2*u_c_* in the measured *d*-spacings was calculated from the standard deviations in the camera constant and in the measurement of the diameters of the experimental rings. The mean and the standard deviation of the camera constant were determined from five measurements each of the diameters of the first seven rings in a SAD pattern from a microcrystalline aluminum standard. The mean and standard deviation of the SAD rings of the experimental sample were determined from three to five measurements of the diameters.

## Footnotes

2 Certain commercial equipment, instruments, or materials are identified in this paper to foster understanding. Such identification does not imply recommendation or endorsement by either the University of Virginia (UVA) or the National Institute of Standards and Technology (NIST), nor does it imply that the materials or equipment identified are necessarily the best available for the purpose.

3 The quoted uncertainty is an expanded uncertainty *U* = *ku_c_*, with *U* determined from a combined standard uncertainty (i.e., estimated standard deviation) *u*_c_ = 2 μm and a coverage factor *k* = 2. The uncertainty reported here is the result of five independent measurements. Since it can be assumed that the possible estimated values of the standard are approximately normally distributed with approximate standard deviation *u*_c_, the unknown value of the standard uncertainty is believed to lie in the interval defined by ± *U* with a level of confidence of approximately 95 %. All uncertainties reported elsewhere in this communication are to be interpreted in this same manner.

4 The mean and the expanded uncertainty values are for 4 and 10 independent measurements, respectively.

5 No statistics were reported in Ref. [9].

6 The sum of the residuals was 20 % smaller when three peaks were considered instead of two, thus indicating that the three-peak profile was more accurate. The expanded uncertainty *U* = 2*u*_c_ in this and subsequent XRD peak measurements is calculated from the standard deviation (*u*_c_), which itself is calculated by the Diffrac 5000 software [7].

7 No statistics were reported in Ref. [13].

8 No statistics were reported in Ref. [10].

9 The ranges indicated for the tungsten and carbon compositions correspond to the experimental deviation of the mean in the EDS measurement of the tungsten and the resulting uncertainties in the carbon calculation.

10 No statistics are reported in Ref. [10].

11 No statistics were reported in Ref. [10].

12 Mean and expanded uncertainty determined from five measurements each in the corresponding FFT images of the lattice fringes of the silver standard and the alloy.

13 No statistics were given in Ref. [10].
